# Evaluation and improvement of LAMP assays for detection of *Escherichia coli* serogroups O26, O45, O103, O111, O121, O145, and O157

**DOI:** 10.4314/ahs.v17i4.8

**Published:** 2017-12

**Authors:** Deguo Wang

**Affiliations:** Henan Postdoctoral Research Base, Food and Bioengineering College, Xuchang University, Xuchang 461000, China

**Keywords:** Loop-mediated Isothermal Amplification (LAMP), toxin-producing *Escherichia coli* serogroups, non-specific amplification, tetramethylene sulfoxide, dimethyl sulfoxide

## Abstract

*Escherichia coli* serogroups O26, O45, O103, O111, O121, O145, and O157 are the causative agents of human diseases, and LAMP assays have been developed for detection of the seven leading STEC serogroups.

**Objective:**

To evaluate existing LAMP assays for detection of the seven STEC serogroups, if necessary, to improve these assays and to promote their practical application.

**Methods:**

Pure DNA extract from 23 strains reserved in our lab was used to evaluate the existing LAMP assays. The existing LAMP assays were modified via adding 1% tetramethylene sulfoxide and 5% dimethyl sulfoxide as well as optimizing reaction temperature.

**Results:**

The detection limit of the modified LAMP assays was 0.1–1 pg per reaction, equivalent to 25–250 cfu per reaction, the non-specific amplification can completely be eliminated with optimal amplifying temperature, and the modified LAMP assays can specifically and sensitivly amplify targeted O serogroup-specific gene (wzx or wzy) of any concerned *Escherichia coli* serogroup as commercial kit Isothermal Master Mix did.

**Conclusion:**

In conclusion, the LAMP assays were highly susceptible to non-specific amplification caused by primer dimers, and these modified methods were free of non-specific amplification and can rapidly and reliably detect the seven major Shiga toxin-producing *E. coli* serogroups.

## Introduction

The method is based on the principle of the reaction performed by a DNA polymerase with strand displacement activity and a set of two specially designed inner primers (FIP and BIP) and two outer primers (F3 and B3). LAMP is highly specific for the target sequence because six independent sequences (F1c, F2, F3, B1c, B2 and B3) recognize the target sequence in the initial stage and four independent sequences (F1c, F2, B1c, and B2) amplify the target sequence in the later stage of the LAMP reaction. Under an isothermal condition, the amplification efficiency of the LAMP method is extremely high because of the absence of a ramp time for thermal change, because it is an isothermal reaction. Therefore, the LAMP assay has the advantage of specificity, selectivity and rapidity over other nucleic acid amplification methods such as polymerase chain reaction (PCR)^2^,^3^, nucleic acid sequence based amplification (NASBA)^4^,^5^, strand displacement amplification (SDA)^6^, rolling circle amplification (RCA)^7^ as well as helicase dependent amplification (HDA)^8^. Moreover, Nagamine et al advanced the method by putting forward loop primers (LF and LB) that accelerated the LAMP reaction^9^.

Shiga toxin-producing *Escherichia coli* (STEC) O26, O45, O103, O111, O121, O145, and O157 are the major serogroups responsible for STEC infections worldwide^10^, many LAMP assays have been established for rapid detection of *Escherichia coli* O157^11^–^15^, only Wang et al had established LAMP assays for the rapid and specific detection of seven leading STEC serogroups with O serogroup-specific genes (wzx or wzy) as target sequences^10^. The study was to evaluate these Wang LAMP assays by dint of StepOneTM System, if necessary, to modify them, and to promote popularization and application of detecting Shiga toxin-producing *Escherichia coli* serogroups with LAMP assays.

## Materials and methods

### LAMP primers

The LAMP primers targeting O serogroup-specific genes (wzx or wzy) of STEC serogroups reported by Wang et al are referenced and used in the study^10^, as shown in Table 1, which were synthesized by Integrated DNA Technologies, Coralville, IA.

**Table 1:** LAMP Primers Targeted wzy or wzx of STEC serogroups.

Target (GenBank accession no.)	Primer Name	Sequence (5′-3′)
O26-wzy (AF529080)	O26-F3	GACTATGAAGCGTATGTTGAT
	O26-B3	TCCTGATTTGAACAATGTCAAT
	O26-FIP	ACCGCCTAAATACTTAACACCATAA-TTAATGTCAATGAACTTTATGCC
	O26-BIP	TTCCTTGGGACCACATTCCT-ACATGTAAAGCAGCAAACC
	O26-LF	ACCAGCGATAACCAATCTC
	O26-LB	TACAATACAGTAAGTATACAGCATT
O45-wzy (AY771223)	O45-F3	AATGTCCCCAGGGTTTGT
	O45-B3	TTTAGTCGCTCGCCAAGA
	O45-FIP	AGCGGGCTAATATTAGTAGTCACTC-GTATGCTTCAATTTGGCTGT
	O45-BIP	ACTCTGGGTTTGATTTTTTCACTTC-ATAATTTCATCCAGACGAACG
	O45-LB	TTATTACTCCTGGCAGTATTAATCG
O103-wzx (AY532664)	O103-F3	ACTCAGTGGTGTAGTAACATG
	O103-B3	TCACCTTGATTTTCTGCTGA
	O103-FIP	ATTTGCTATTCCAATTGGACCAGTA-CTTTAGACTAATTTGTGGCCTTC
	O103-BIP	TTGGGACAATTGCAAAATTTTGTGG-ATCTATTAACTCCTTGTGAAACTTG
	O103-LF	AATTGCAACAACTTTTGAAATAA
	O103-LB	CCTTTATAAATGGATTCATTTCATC
O111-wzy (AF078736)	O111-F3	AAGGCGTAACTTTTTTTGAAC
	O111-B3	TCATGAGGGTCATTAGGAATT
	O111-FIP	TCACCAAGCTGTGAAACCAAA-CTACAGCAAGTAATATTGAACGT
	O111-BIP	TCCATGGTATGGGGACATTAAATTT-TGATGGAAGTCCATATAACGT
	O111-LB	CTTAAATAACGGCGGACAAT
O121-wzy (AY208937)	O121-F3	GCTCAGCTTTTATCTTGTTCAA
	O121-B3	ATAGGCTCCCAACCATCC
	O121-FIP	ACGCAAAAAGTATGGATTCATACCT-GATATAACAGAACCGACTTGG
	O121-BIP	TGTTGCTGGTTCCTTATTATGTAGT-AAAAGCAAGCCAAAACACTC
	O121-LF	TAAAGCCATCCAACCACGC
O145-wzx (AY647260)	O145-F3	TTTGTAAGACAAGGTGTATGG
	O145-B3	GCATTGGTACAGACAGCTTTA
	O145-FIP	CACAGTACCACCAAACCAAAAAATA-TTGGTTAGCTATAGCTGTGA
	O145-BIP	AGTGTGCTTGGAGTGGCTTA-CAATCCCAGTTTGTAATATCGC
	O145-LF	TTCTTAAGTTCGGATACACTAGCA
O157-wzy (AF061251)	O157-F3	TCCCTTTAGGGATATATATACCTT
	O157-B3	ATAACTGATATTTTCATTTCGTGAT
	O157-FIP	TTCCCAGCCACTAAGTATTGCAATA-TGAAAAAAACCCATAGCTCGA
	O157-BIP	TGCATCGGCCTTCTTTTTTGG-AACGTATCATGCAATAAGATCA
	O157-LF	ATAATGATATATGAATAGAATGCGC
	O157-LB	TCCTTTTCTCTCCGTATTGAT

### Bacteria strains and DNA extraction

Twenty-three strains were used for the specificity study (Table 2). *Listeria* strains were cultured overnight at 37 °C in DifcoTM Buffered *Listeria* Enrichment Broth Base (Becton, Dickinson and Company) and the others in Luria-Bertani (LB) broth. DNA from these pure cultures was extracted according to the manufacturer's handbook of DNeasy® Blood & Tissue Kit (QIAGEN N.V.), and these DNA templates was used for evaluation of Wang LAMP Assays, Modified LAMP assays as well as commercial Isothermal Master Mix.

**Table 2:** Bacterial Strains Used in the Study.

Bacterial Strain (Serotype)	Bacterial Strain (Serotype)
*Escherichia coli* O121:H19	*Listeria monocytogenes* J1-094 (1/2c)
*Escherichia coli* O26:H11	*Listeria monocytogenes* C1-115 (3a)
*Escherichia coli* O111:H8	*Listeria monocytogenes* J1-031 (4a)
*Escherichia coli* O145:H2	*Listeria monocytogenes* W1-110 (4c)
*Escherichia coli* O103:H2	*Listeria monocytogenes* ATCC19115 (4b)
*Escherichia coli* O45:H12	*Listeria innocua* ATCC51742
*Listeria monocytogenes* J1-225 (4b)	*Listeria invanovii* ATCC49954
*Listeria monocytogenes* J2-020 (1/2a)	*Salmonella typhimuriam*
*Listeria monocytogenes* J2-064 (1/2b)	*Salmonella enterica* serotype Newport
*Listeria monocytogenes* J1-169 (3b)	*Escherichia coli* O157:H7 933
*Listeria monocytogenes* J1-049 (3c)	*Escherichia coli* O157:H7 B1409
*Listeria monocytogenes* M1-004 (N/A)	Reserved in our lab

### Sensitivity Determination of Wang LAMP Assays

The LAMP was carried out in a total 25 mL reaction mixture containing 1× ThermolPoly reaction buffer (New England Biolabs, Beverly, Mass., U.S.A.), 6 mM MgSO_4_, 1.2 mM each deoxynucleoside triphosphate (dNTP), 0.1 µM F3 and B3, 1.8 µM FIP and BIP, 1.0 µM LF and LB, 10 units of Bst 2.0 WarmStart DNA polymerase (New England Biolabs), 1×EvaGreen® dye (Biotium, Inc.), 1×Reference Dye for Quantitative PCR (Sigma), and serial dilutions of DNA template of concerned STEC serogroup ranging from 100–1 pg according to the report of Wang, et al^10^. Two positive controls and three negative controls were included in each LAMP run. LAMP reaction mixtures were heated at 65°C (63°C for *E. Coli* O157) for 90 min in StepOneTM System (30 sec/cycle); the amplification had been extended from reported 50 min to 90 min in order to overall evaluate the amplification.

### Temperature Optimization of Modified LAMP Assays

Upon the results of evaluation, Wang LAMP assays were modified by adding 1% tetramethylene sulfoxide and 5% dimethyl sulfoxide into LAMP reaction mixture with 100 pg concerned DNA template. The reaction mixture was pre-heated at 95°C for 5 min before Bst 2.0 WarmStart DNA polymerase was added, and then the LAMP reaction mixture was heated at 65°C, 63°C, 61°C, 59°C, 57°C, 55°C and 53°C for 50 min in StepOneTM System, respectively.

### Sensitivity comparison of modified LAMP assays and isothermal master mix

The modified LAMP mixture with serial dilutions of DNA template of concerned STEC serogroup ranging from 10–0.01 pg was heated at optimal temperature for 50 min in StepOneTM System, and the detection limits of modified LAMP assays were determined.

For comparison, Isothermal Master Mix purchased from OptiGene Limited, and the LAMP reaction was carried out according to the manufacturers' instructions using same LAMP primers as modified LAMP Assays with serial dilutions of DNA template of concerned STEC serogroup ranging from 10–0.1 pg.

### Specificity determination of modified LAMP assays and isothermal master mix

Twenty-three strains were used for the specificity study on modified LAMP assays and Isothermal Master Mix (Table 2), and the amount of DNA template used is 100 pg per reaction.

## Results and analysis

### Defect of Wang LAMP assays found in sensitivity determination

**Figure 1:**
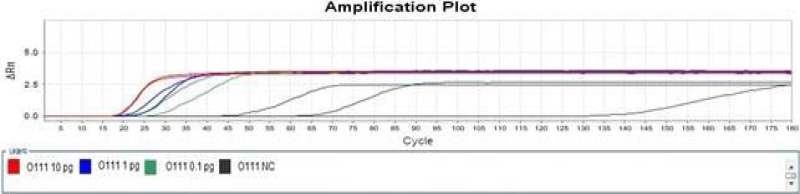
Amplification Plot of Wang LAMP assay for detection of *E. coli* O111.

**Figure 2:**
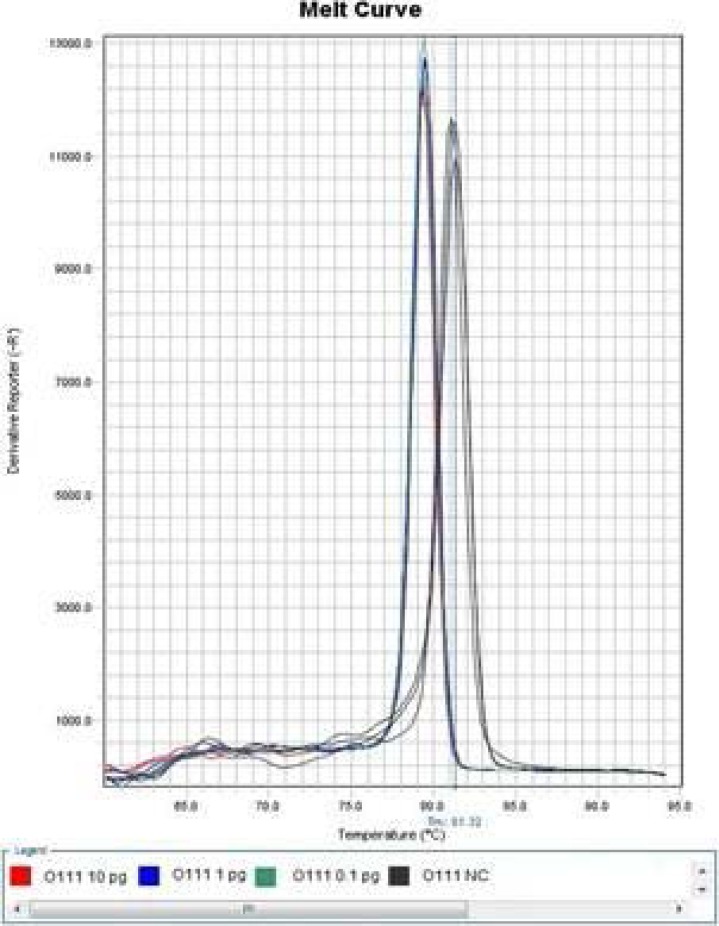
Melt Curve of modified LAMP assay for detection of *Escherichia coli* O111 at 57 °C.

The sensitivity determination results of Wang LAMP assays were summarized in Tab 3, the amplification after 50 min was judged as negative in order to keep fairly comparison with report of Wang et al in 2012^10^, our experiment indicated that it was difficult to differentiate specific amplification from non-specific amplification just via the amplification plot, but the melt curve of specific amplification was significantly different with that of non-specific amplification (Figure 1 and Figure 2. Only the amplification plot and melt curve of LAMP Assay for determination of *Escherichia coli* O111 were given in this paper due to space limitations.

**Table 3:** Sensitivity determination of Wang LAMP assays.

Gene	Specific or Non-specific Amplification	100 pg	10 pg	1 pg	NC
O111-wzy	Specific	2/2	2/2	2/2	0/3
	Non-specific				2/3
O45-wzy	Specific	2/2	2/2		
	Non-specific			2/2	3/3
O26-wzy	Specific	2/2	2/2		
	Non-specific			2/2	3/3
O145-wzx	Specific	2/2	2/2		
	Non-specific			2/2	3/3
O103-wzx	Specific	2/2	2/2	0/2	0/3
	Non-specific			0/2	1/3
O157-wzy	Specific	2/2	2/2		0/3
	Non-specific			2/2	1/3
O121-wzy	Specific	2/2	2/2	2/2	0/3
	Non-specific				2/3

Wang et al performed LAMP reaction in an LA-320C Real-time Turbidimeter (Eiken Chemical Co., Ltd., Tokyo, Japan) 10, it was also difficult to discover non-specific amplification only according to turbidity curve. In contrast, real-time PCR instrument was the most suitable tool in study of nucleic acid amplification to a large extent. Non-specific amplification can be differentiated from specific amplification by aid of real-time PCR instrument, and then the cause (non-specific amplification or aerosol pollution) of false positive can be confirmed. It was verified by the sensitivity experiment that Wang LAMP assay for any of concerned *Escherichia coli* serogroups had the defect of non-specific amplification, as Table 3, Figure 1 and Figure 2 indicated.

### Temperature optimization of modified LAMP assays

1% tetramethylene sulfoxide and 5% dimethyl sulfoxide were added to the reaction mixtures of Wang LAMP Assays, and the reactions were carried out at varying temperatures for 50 min. The optimal temperature were selected according to threshold, but the difference of the thresholds at 57°C and at 55°C in detection of *Escherichia coli* O111 as well as that of the thresholds at 59°C and at 57°C in detection of *Escherichia coli* O121 was not obvious, the amplification temperature for detection of *Escherichia coli* O111 and *Escherichia coli* O121 was to be further optimized in the sensitivity determination of modified LAMP assays, as Table 4 indicated.

**Table 4:** Sensitivity determination of modified LAMP assays and isothermal master mix.

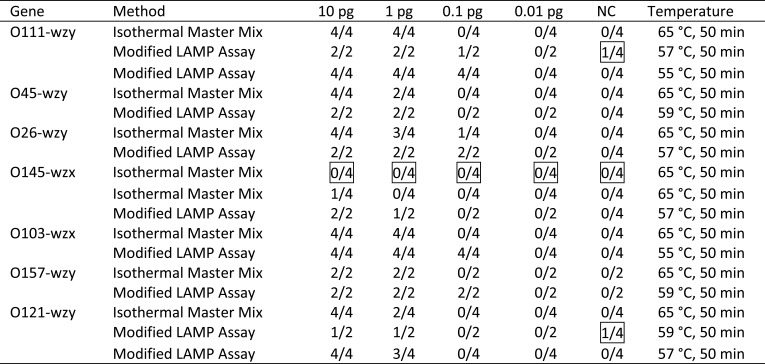

### Sensitivity comparison of modified LAMP assays and isothermal master mix

**Figure 3:**
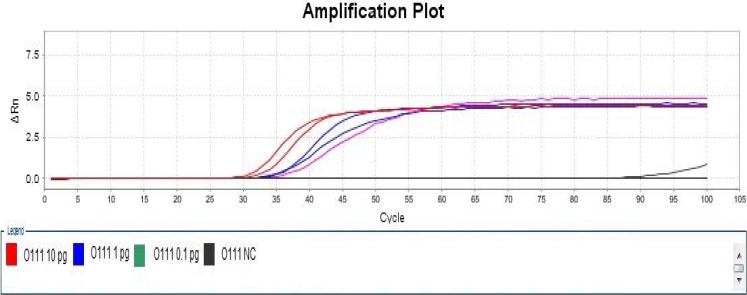
Amplification Plot of Modified LAMP Assay for Detection of *Escherichia coli* O111 at 57 °C.

**Figure 4:**
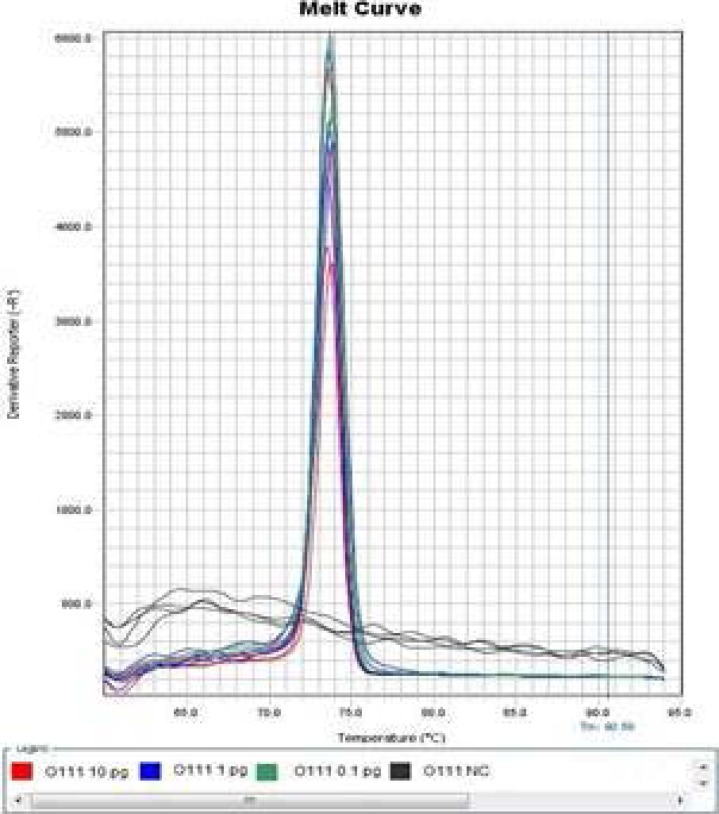
Melt Curve of Modified LAMP Assay for Detection of *Escherichia coli* O111 at 57 °C.

**Figure 5:**
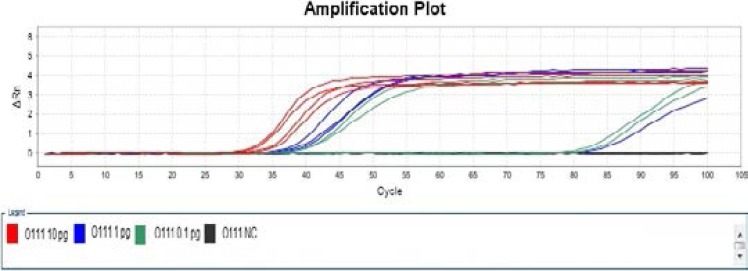
Amplification Plot of Modified LAMP Assay for Detection of *Escherichia coli* O111 at 55 °C

**Figure 6:**
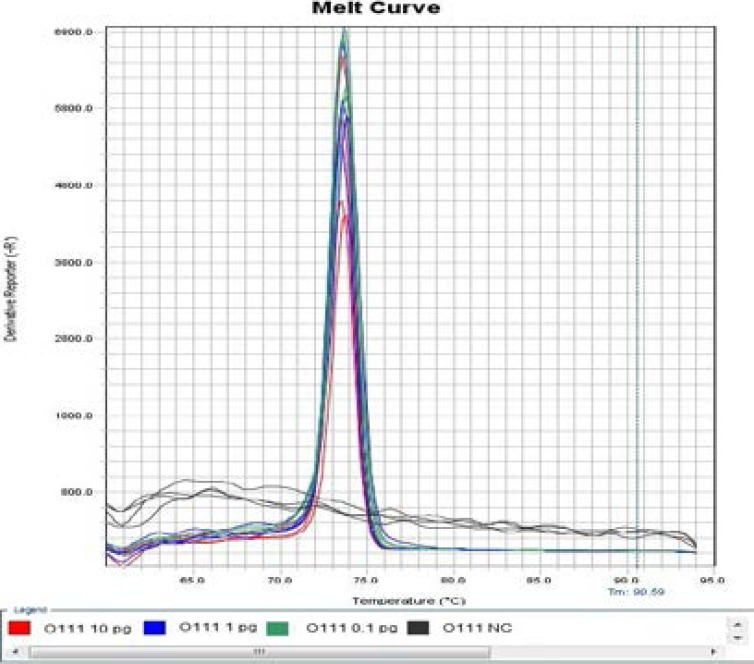
Melt Curve of Modified LAMP Assay for Detection of *Escherichia coli* O111 at 55 °C

As far as the modified LAMP assays were concerned, one of four negative controls in detection of *Escherichia coli* O111 had non-specific amplification at 57°C (Figure 3 and Figure 4), two of two Positive Controls with 0.1 pg DNA template, one of two Positive Controls with 0.01 pg DNA template as well as one of four Negative Controls in detection of Escherichia coli O121 had non-specific amplification at 59°C, while the reactions at 55°C and 57°C, respectively, did not have non-specific amplification (Figure 5 and Figure 6), therefore, temperature was one of critical factors having significant effect on specific or non-specific amplification of the modified LAMP Assays.

At the selected temperature, the modified LAMP assays can specifically amplify targeted O serogroup-specific gene (wzx or wzy) of any concerned *Escherichia coli* serogroup, the detection limits ranged from 0.1 pg to 1 pg DNA templates per reaction mixture, as Table 4 shows.

**Figure 7:**
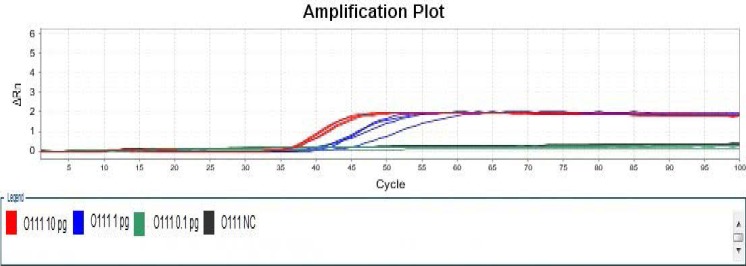
Amplification Plot of Isothermal Master Mix for Detection of *Escherichia coli* O111.

**Figure 8:**
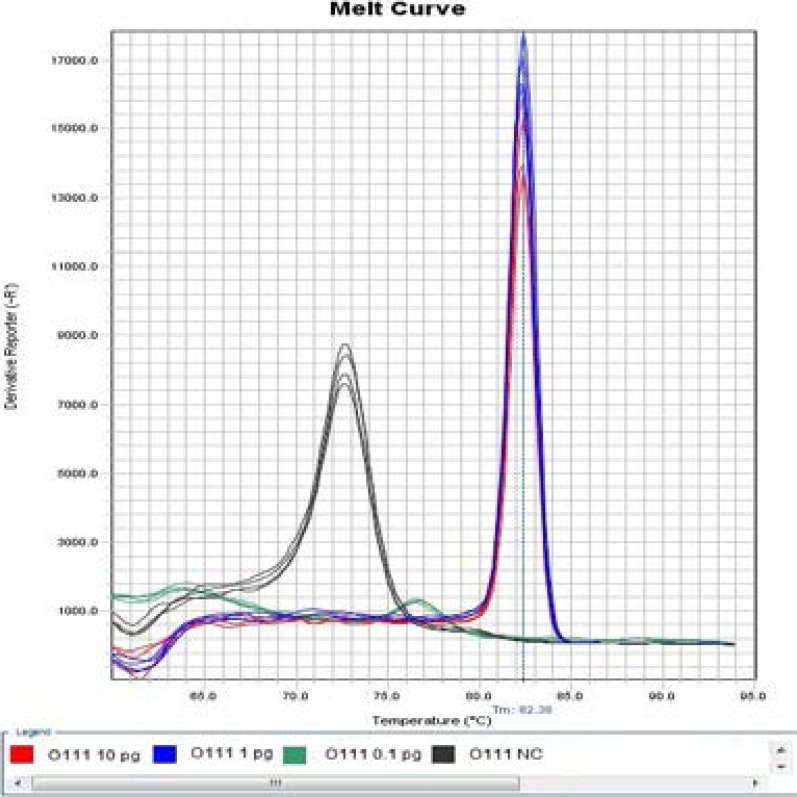
Melt Curve of Isothermal Master Mix for Detection of *Escherichia coli* O111.

Judged according to amplification plots as well as melt curves, with the amplification plot and melt curve of *Escherichia coli* O111 given in this paper as example (Figure 7 and Figure 8), the Isothermal Master Mix can specifically detect seven major Shiga toxin-producing *E. coli* serogroups, the detection limits of the Isothermal Master Mix using same LAMP primers as the modified LAMP Assays were obviously higher than those of the modified LAMP assays, especially for *Escherichia coli* O145. All of the reactions including 4 positive controls with 10 pg DNA templates of *Escherichia coli* O145 were negative, for further verification, the experiment was repeated, only one of 4 positive controls with 10 pg DNA templates of *Escherichia coli* O145 was positive, while the modified LAMP assay can detect one of two positive controls with 1 pg DNA templates of *Escherichia coli* O145, as Table 4 indicated.

### Specificity determination of modified LAMP Assays and isothermal master Mix

Due to the non-specific amplification found in sensitivity determination experiment, the specificity of Wang LAMP assays was no longer tested with bacterial strains. The modified LAMP assays and the Isothermal Master Mix were tested with 23 bacterial strains (Tab 2) and with TE buffer as negative controls, 4 repeats per reaction. The results indicated that both the modified LAMP assays and the Isothermal Master Mix can specifically detect concerned *Escherichia coli* serogroup, while the detection reactions of other bacteria and negative controls were negative.

## Discussion

Real-time PCR instrument is a versatile tool for study on amplification of nucleic acids, it can detemine the melt cure of amplified product for judgement of non-specific amplification, and it had been used to evaluate Wang LAMP assays in the study. Either non-specific amplification or aerosol contamination can result in false positive results of LAMP, but, as our experiment indicated, the melt curve of specific amplification was significantly different with that of non-specific amplification, therefore, the non-specific amplification can be distinguished from that of aerosol contamination, It was found via the amplification plots and melt curves that all Wang LAMP assays had the defect of non-specific amplification.

It is apt to non-specifically amplify when couple numerous sets of high-concentration primers are used in LAMP assays. This is especially true when the concentrations of primers, Mg^2+^, dNTPs and DNA Polymerase in reaction mixtures are many times as high as those used in Real-time PCR. The concentrations of these 4 factors must be strictly controlled to avoid non-specific amplification in real-time PCR^16^. There are instances in which standard PCR amplification reaction conditions do not produce acceptable results. Addition of tetramethylene sulfoxide and dimethyl sulfoxide has been used improve PCR results^17^–^20^. We investigated these approaches for the first time for optimization of LAMP reactions. It is fair to state the potential effect of 1% tetramethylene sulfoxide and 5% dimethyl sulfoxide in realization of the specific amplication of all Wang LAMP assays for detection of all concerned STEC serogroups at optimal temperature.

## Conclusion

In summary, we had found that all Wang LAMP assays for detection of 7 main STEC serogroups had the defect of non-specific amplification by aid of Real-time PCR instrument, and we had improved these methods via adding 1% tetramethylene sulfoxide and 5% dimethyl sulfoxide into LAMP reaction mixtures as well as optimizing temperature. These modified LAMP assays can sensitively and specifically detect corresponding main STEC serogroups as commercial Isothermal Amplification Kit does.
